# First *in Vivo Batrachochytrium dendrobatidis* Transcriptomes Reveal Mechanisms of Host Exploitation, Host-Specific Gene Expression, and Expressed Genotype Shifts

**DOI:** 10.1534/g3.116.035873

**Published:** 2016-11-16

**Authors:** Amy R. Ellison, Graziella V. DiRenzo, Caitlin A. McDonald, Karen R. Lips, Kelly R. Zamudio

**Affiliations:** * Department of Ecology and Evolutionary Biology, Cornell University, Ithaca, New York 14853; † Department of Biology, University of Maryland, College Park, Maryland 20742

**Keywords:** *Batrachochytrium dendrobatidis*, laser capture microdissection, transcriptomics, gene expression, pathogen

## Abstract

For generalist pathogens, host species represent distinct selective environments, providing unique challenges for resource acquisition and defense from host immunity, potentially resulting in host-dependent differences in pathogen fitness. Gene expression modulation should be advantageous, responding optimally to a given host and mitigating the costs of generalism. *Batrachochytrium dendrobatidis* (*Bd*), a fungal pathogen of amphibians, shows variability in pathogenicity among isolates, and within-strain virulence changes rapidly during serial passages through artificial culture. For the first time, we characterize the transcriptomic profile of *Bd in vivo*, using laser-capture microdissection. Comparison of *Bd* transcriptomes (strain JEL423) in culture and in two hosts (*Atelopus zeteki* and *Hylomantis lemur*), reveals >2000 differentially expressed genes that likely include key *Bd* defense and host exploitation mechanisms. Variation in *Bd* transcriptomes from different amphibian hosts demonstrates shifts in pathogen resource allocation. Furthermore, expressed genotype variant frequencies of *Bd* populations differ between culture and amphibian skin, and among host species, revealing potential mechanisms underlying rapid changes in virulence and the possibility that amphibian community composition shapes *Bd* evolutionary trajectories. Our results provide new insights into how changes in gene expression and infecting population genotypes can be key to the success of a generalist fungal pathogen.

The success of pathogens depends on their ability to invade, colonize, and reproduce within host tissues. Therefore, pathogens require finely tuned expression of genes during the dynamic infection process, allowing them to counter or evade host defense responses, and acquire nutrients to invest in growth and reproduction. For generalist pathogens, each host species represents a distinct selective environment, providing host-specific defense mechanisms to counter, and unique conditions for resource acquisition (Ellison *et al.* 2015), potentially contributing to observed differences in pathogen fitness on different hosts. Thus, modulation of gene expression should be advantageous to generalist pathogens, responding optimally to a given host and mitigating costs of generalism (Leggett *et al.* 2013). To gain insight into the mechanisms of within-host survival, host differences in pathogen virulence, and how the infection process shapes generalist pathogen evolution, we require characterization of gene expression *in vivo* in a diversity of hosts.

The study of many pathogenic microbes necessitates their maintenance in the laboratory using artificial culturing media—a far cry from the complex and often hostile environment of a living host. *In vitro* culturing of pathogens can result in drastic alterations in phenotypes (Franzot *et al.* 1998; Vandenberg and Cantone 2004), genotypes (Somerville *et al.* 2002; Garcia-Hermoso *et al.* 2004; Farrer *et al.* 2013), and, importantly, pathogenicity (Brummer *et al.* 1990; Hess *et al.* 2008; Safavi 2012). These changes undoubtedly involve underlying shifts in gene expression (La *et al.* 2008; Zbell *et al.* 2008; Gibbons *et al.* 2012), and possibly in the proportion of genotypes represented in pathogen populations. Culture media, while providing the necessary nutrients and conditions to grow and reproduce pathogens, is a simpler or more relaxed selective environment, potentially resulting in sequence or expressed genotypes highly divergent to those seen in natural hosts.

*Batrachochytrium dendrobatidis* (*Bd*) is a generalist fungal pathogen capable of infecting hundreds of host species (Skerratt *et al.* 2007; Olson *et al.* 2013), and is a major cause of global amphibian decline (Berger *et al.* 1998; Lips *et al.* 2006). The pathogenicity of *Bd* varies among isolates and phylogenetic lineages (Berger *et al.* 2005; Fisher *et al.* 2009; Farrer *et al.* 2011), and within-strain virulence can change rapidly during serial passages through artificial culture conditions. However, the underlying mechanisms responsible for variation in pathogenicity to hosts and rapid shifts in virulence are not fully understood (Rosenblum *et al.* 2013; Voyles *et al.* 2014). The environment in which *Bd* grows—including both abiotic factors and host skin conditions—is likely crucial to strain differences in pathogenicity and virulence.

*Bd* is successfully maintained in the laboratory in tryptone broth and on agar plates (Longcore *et al.* 1999), where successive passages through culture often leads to attenuation (Berger *et al.* 2005; Retallick and Miera 2007; Langhammer *et al.* 2013; Voyles *et al.* 2014). Furthermore, previously attenuated strains can regain pathogenicity after a single passage through an amphibian host (Brem *et al.* 2013). Though short-term evolutionary processes such as rapid changes in ploidy levels (Farrer *et al.* 2013; Rosenblum *et al.* 2013; Refsnider *et al.* 2015), relative abundances of intrastrain genotypes, and/or gene expression regulation are promising explanations, it is still unclear how the virulence and pathogenicity of this infectious pathogen can evolve so rapidly.

Simultaneously capturing the full extent of host and pathogen transcriptomes has, until recently, been a technical challenge, in large part due to the often very low ratios of pathogen mRNA within infected host tissue. However, techniques to isolate infection at the cellular level (Datta *et al.* 2015), coupled with development of methods to capture whole-transcriptomic data from ultralow mRNA quantities (even at the single-cell level; Tang *et al.* 2009), now enables us to observe host–pathogen interactions at a new level of detail. Here, we apply these techniques to capture the transcriptomes of *Bd*, the amphibian-killing fungus, within living hosts.

Our goal in this study was to characterize *in vivo* transcriptomes of *Bd* infections growing within two highly susceptible amphibian host species, and to compare expression profiles with those derived from *Bd* in culture. With these first *in vivo* transcriptomes, we address the following questions: (1) does gene expression differ between *Bd* in culture and in amphibian skin, and can these differences be linked to biological processes related to host exploitation? (2) Does host species influence *Bd* gene expression? And (3) do we detect significant changes in *Bd* genotype frequencies between culture and host infections? Taken together, answers to these questions elucidate mechanisms of *Bd* host exploitation, and how shifts in virulence can occur so rapidly in this fungal pathogen.

## Materials and Methods

### Experimental infections and tissue harvesting

Four captive-bred adult *Atelopus zeteki* (41 months postmetamorphosis) and *Hylomantis lemur* (12 months postmetamorphosis) were used for experimental infections. Prior to experiments, frogs of each species were cohoused, had never been exposed to or tested positive for *Bd*, and never been treated with antifungals. All individuals appeared healthy and tested negative for *Bd* at the start of the experiment. Animals were housed individually in plastic shoeboxes with a water dish and paper towel, in a laboratory maintained at 21–22° with a 12:12 hr light:dark photoperiod. All housing materials were replaced every 7 d, water dishes changed every 3 d, and animals were fed crickets and fruit flies sprinkled with Herptivite (Rep-Cal Research Labs) every 3 d.

For experimental infections, we used *Bd* strain JEL423, cultured (Longcore *et al.* 1999) and cryopreserved from a field-infected *H. lemur*, during the 2004 epidemic outbreak at El Copé, Panama (Lips *et al.* 2006). We inoculated each experimental animal for 10 hr in a 50 ml bath of *Bd* zoospores (2 × 10^4^ zoopsores/ml). To provide culture-controls of the same passage number (total five passages), the same zoospore stock was used to inoculate four agar plates (1 ml at 1 × 10^5^ zoospores/ml) and cultured at 21–22° for 7 d. This ensured all frogs and plates were inoculated with an equivalent number zoospores (1 million). After culturing, *Bd* colonies were scraped from plates using sterile instruments, twice syringe-filtered (Life Sciences Acrofilter 10 µm membrane) to obtain sporangia, pelleted by centrifugation, snap-frozen using liquid nitrogen, and stored at −80° until RNA extraction.

Throughout the infection trial, we used a fresh pair of latex powder-free gloves when handling each individual frog. All individuals were assayed for *Bd* infection status and load at d 8 postinoculation, and thereafter every 3–4 d. We swabbed the abdomen, drink patch, hands, and feet, five times each with a sterile cotton tipped swab, which was then stored in capped tubes containing 30 μl of 70% ethanol (Hyatt *et al.* 2007). We tested swabs for *Bd* using PrepMan Ultra extractions and singlicate Taqman quantitative polymerase chain reaction (qPCR) (Boyle *et al.* 2004). We ran each plate with JEL423 standards of 0.1, 1, 10, 100, and 1000 zoospore genomic equivalents to determine *Bd* presence and infection intensity. We categorized individuals as *Bd*-positive when qPCR results showed an infection load ≥1 *Bd* zoospore genomic equivalent (Kriger *et al.* 2006). Individuals were monitored daily for clinical signs of chytridiomycosis, and killed by application of 10% Benzocaine to the venter once they lost righting abilities.

Immediately after euthanasia, using sterile instruments, skin from the ventral thigh was harvested from each individual. Skin pieces were placed in Tissue-Tek cryomolds (Sakura Finetek), covered in Tissue-Tek Optimum Cutting Medium (OCT, Sakura Finetek), and immediately snap-frozen in an isopentane/dry ice bath. Molds were stored at −80° until cryosectioning. As time to death due to Benzocaine treatment was rapid (<2 min), and skin tissue was harvested away from site of application, we minimized the potential for the euthanasia method to affect *Bd* gene expression, and therefore confound comparison with cultured *Bd*.

The number of *Bd* zoospores that infect individuals during inoculations is highly variable, causing natural differences in infection intensities and time to death among individuals and species (Carey *et al.* 2006). In this study, clinical signs of chytridiomycosis developed 15–18 d postinoculation in *A. zeteki* and 35–51 d in *H. lemur*. We cannot ascertain that all frogs were at the exact same stages of disease progression at the time of sampling. However, the infected frogs and culture plates used in this study had in common that all were at a “mature” infection stage, colonized with mature zoosporangia and shedding zoospores. At the point of tissue sampling, all frogs carried their highest individual *Bd* load, and thus all could be considered still within the disease progression phase.

### RNA extraction and sequencing

To minimize RNA degradation, all sectioning, staining, and laser-capture microdissection (LCM) steps were performed as quickly as possible. Prior to cryosectioning, PEN membrane slides (Zeiss) were sterilized by dipping in RNase Zap (Ambion) for 30 sec, rinsed in nuclease-free water, and dried for 30 min under UV lights. Tissue molds were allowed to equilibrate to −20° within the cryostat for ∼15 min prior to sectioning. Approximately five 8 µm skin sections were mounted on each prechilled slide. Slides were fixed in ice-cold 100% ethanol for 5 min, placed individually in sterile 50 ml falcon tubes containing desiccant, and stored at −80° (for a maximum of 4 wk) until LCM. Cryostat blades were changed between each sample.

Immediately before LCM, slides were removed from −80° and placed in ice-cold nuclease-free water for 30 sec to remove OCT. To aid rapid identification of highly infected skin areas, slides were placed for 30 sec in Calcofluor white (1%, Sigma-Aldrich)—a chitin-binding fluorescent stain. Slides were then rinsed in ice-cold nuclease-free water, and dehydrated for 30 sec in 70%, 75%, 95%, and 1 min in 100% ice-cold ethanol. Stained and dehydrated slides were immediately microdissected using a Zeiss PALM MicroBeam laser capture system with a 430 nm filter. For each slide, we dissected as many infected areas of the stratum corneum and the immediately underlying epidermal cells as possible within 2 hr. Previous studies of RNA integrity during LCM indicate that dehydrated slides can be worked on for up to 4 hr without significant loss of RNA quantity or quality (Grover *et al.* 2012). Microdissected skin areas were collected in 500 µl adhesive-capped tubes (Zeiss). After collection, tissue was lysed by addition of 120 µl of Qiagen RLT buffer with β-mercaptoethanol (10 µl in 1 ml buffer), vortexed for 30 sec, and incubated at room temperature in an upside-down position for 30 min. Tubes were then centrifuged for 5 min, and stored at −80° until RNA extraction.

For each individual, three lysates were pooled, and total RNA extracted using RNeasy micro kits (Qiagen) and eluted in 15 µl nuclease-free water. Due to the extremely low quantities of RNA (typically undetectable using high-sensitivity RNA Qubit assays; <0.2 pg/µl), whole transcriptome amplification was performed prior to Illumina sequencing library preparation. cDNA reverse transcription and amplification was performed using Clontech SMARTer Ultra Low RNA Amplification kit using 5 µl eluted total RNA, and the minimum number of PCR cycling steps (nine cycles). Amplified cDNA was quantified using Qubit high-sensitivity DNA assays. Sequencing libraries were prepared and uniquely indexed using the Illumina Nextera XT kit with 100–150 pg input cDNA. Libraries were assessed for quality and fragment size range using Bioanalyzer 2100 assays. SMARTseq whole transcriptome amplification is demonstrated to have negligible systematic biases, particularly in multi-cell preparations as used here (Ramsköld *et al.* 2012). However, identical RNA extraction and mRNA amplification protocols were used for cultured *Bd* to avoid potential biases between the culture and *in vivo Bd* transcriptomes.

Libraries were pooled and sequenced using Illumina NextSequation 75 bp runs. Pilot sequencing studies indicated that six frog/*Bd* samples per lane (∼60 million reads per sample) would provide sufficient sequencing depth to fully capture the transcriptomes of both host and *Bd*. Cultured *Bd* libraries were sequenced at a sequencing depth equivalent to 16 samples per lane (20 million reads per sample) because only *Bd* gene expression would be captured in these samples (and thus would not be dominated by host transcripts).

### Gene expression analyses

After Illumina standard quality control filtering (all reads flagged as low quality were removed), read quality for each sample was visualized using FastQC version 0.10.0 (Andrews 2010), checking for per base sequence quality, GC content, and overrepresented sequences (*e.g.*, contamination). We used Trimmomatic version 0.27 (Lohse *et al.* 2012) to: (1) trim Illumina adapter sequence, (2) trim the 5′ and/or 3′ end of reads where quality score dropped below Q20, (3) trim anywhere within each read where a 5 bp window dropped below Q20, and (4) discard any trimmed reads <36 bp long. This ensured only the highest quality reads were used for subsequent analyses. For each *A. zeteki* sample, reads were separated into those derived from frog and *Bd* by first mapping all reads to the published JEL423 transcriptome (*Batrachochytrium dendrobatidis* Sequencing Project, Broad Institute) using bowtie v2.2.4 with default parameters (Langmead *et al.* 2009). Unmapped reads were then mapped to a previously assembled *A. zeteki* transcriptome (Ellison *et al.* 2014). To further ensure read identity, we removed all reads that mapped to both host and pathogen transcriptomes. Gene expression raw read counts were obtained for both host and pathogen using RSEM v1.2.2 (Li and Dewey 2011). As *H. lemur* has no available transcriptome, reads were first mapped to the *Bd* transcriptome, and then unmapped reads were pooled from all samples and *de novo* assembled into *H. lemur* transcripts using previously published pipelines utilizing the Trinity assembly package (Haas *et al.* 2013; Ellison *et al.* 2014, 2015).

To compare *Bd* gene expression between culture and the two host species, differential expression analyses were performed using DESeq2, which normalizes raw read counts by library depth (Love *et al.* 2014). Genes were deemed significantly differentially expressed using a false discovery rate (FDR) corrected p-value of 0.05. Due to the potentially variable degree of transcriptome coverage resulting from the within-host samples, only genes that had detectable expression in at least two samples of all three conditions (culture, *A. zeteki* host, and *H. lemur* host) were considered for analysis (8268 of the 8819 JEL423 reference transcripts). Functional annotation [gene ontology (GO) mapping and InterPro scans] of the JEL423 reference transcriptome was performed using Blast2Go (Conesa *et al.* 2005), using default settings (Supplemental Material, File S4). GO enrichment tests (with FDR correction) were carried out on each group of differentially expressed genes via Blast2Go to detect significantly over-represented biological processes and molecular functions. Similarity of per-sample *Bd* gene expression profiles was visualized using hierarchical clustering of Poisson distances of gene expression values using DESeq2.

### Expressed single nucleotide variant (eSNV) analyses

*Bd* sequence reads for each treatment (culture, *A. zeteki* or *H. lemur*) were pooled, and single nucleotide variants (SNV) were called using samtools mpileup using default settings (Li *et al.* 2009). RNAseq data do not allow us to discriminate whether SNV allele frequency differences are due to allele-specific expression, or underlying genotype frequency differences in pooled populations (Konczal *et al.* 2014); therefore, we refer only to “expressed genotypes.” We used the program Popoolation2 (Kofler *et al.* 2011a) to calculate expressed SNV (eSNV) allele frequencies within each treatment pool. We applied the conservative parameters of minimum mapping quality = 20, minimum count = 6, and minimum coverage = 20 (Kofler *et al.* 2011b), and also tested a higher minimum allele count of 10 to ensure the results were robust (Figure S1). To further filter out potential sequencing errors, only eSNVs with a minor allele frequency of ≥0.1 were used in subsequent analyses. These criteria allowed investigation of differences in eSNV allele frequencies between treatment populations, but not detection of newly acquired SNVs, as each polymorphic site had to be detected in all populations (*i.e.*, in culture and both host species) to be considered further. Significant differences in eSNV allele frequencies were determined using two methods. First, Fisher’s exact tests (and FDR-corrected p-values) were used to estimate the significance of eSNV allele frequency differences between treatment pools. Second, each sample was treated as a separate, replicate pool of *Bd* and tested in pairs using the Cochran-Mantel-Haenszel test implemented in Popoolation2. Only biallelic eSNVs found to be significant in both analyses were considered further. Custom Perl scripts were used to determine if SNVs were synonymous or nonsynonymous. Blast2Go enrichment analyses were used to test for GO term enrichment in sets of genes containing eSNVs found to have significant differences in allele frequencies between treatment groups.

### Data availability

All sequence data are available at the NCBI Short Read Archive with the accession number SRP064950. File S1 contains details of all differential gene expression tests. File S2 contains all eSNVs with significant differences in frequencies and their locations. File S3 contains details of the eSNVs matched to previous resequencing studies. File S4 contains the GO annotation of the JEL423 reference transcriptome generated by Blast2Go. File S5 contains the custom perl script used to determine synonymous and nonsynonymous eSNVs. Table S1 and Table S2 contain the top 20 differentially expressed Bd genes of all comparisons between culture plates and amphibian hosts. Figure S1 shows sample separation by eSNV frequencies using two different minimum allele count thresholds and when considering only SNVs found in previous resequencing studies. A Venn diagram of the overlap of genes found to have significant differential expression and eSNV frequencies is provided in Figure S2. 

## Results

### Host and Bd transcriptome recovery

All experimental animals were killed upon exhibiting severe signs of chytridiomycosis (*i.e.*, loss of righting reflex). At time of tissue sampling, *Bd* zoospore loads of *A. zeteki* ranged between 23.44 and 174.75 thousand zoospore genomic equivalents (K ZGE). *H. lemur* loads ranged between 0.40 and 122.68 K ZGE (Table 1).

**Table 1:** Summary of sample infection loads, read mapping, and *Bd* gene detection

ID	Host	*Bd* Load (K ZGE)	No. Mapped Reads (M)	% Mapped Read		No. *Bd* Genes	% *Bd* Genes
				Bd	Host		
C1	Culture	—	19.85	100.00	—	7782	94.12
C2	Culture	—	15.54	100.00	—	7763	93.89
C3	Culture	—	19.70	100.00	—	7766	93.93
C4	Culture	—	18.18	100.00	—	7791	94.23
AZ1	*A. zeteki*	89.62	48.19	34.65	65.35	7315	88.47
AZ2	*A. zeteki*	23.44	35.73	39.90	60.10	7932	95.94
AZ3	*A. zeteki*	174.75	23.66	24.79	75.21	7823	94.62
AZ4	*A. zeteki*	44.77	65.01	12.69	87.31	7678	92.86
HL1	*H. lemur*	20.95	59.28	8.15	91.85	7243	87.60
HL2	*H. lemur*	11.09	52.48	6.70	93.30	7329	88.64
HL3	*H. lemur*	122.68	44.77	4.03	95.97	6284	76.00
Total	—	—		—	—	8268	100.00

K ZGE, thousand zoospore genomic equivalents.

Of the 8819 genes in the published JEL423 genome, a total of 8268 was detected in at least one sample (*i.e.*, with one or more uniquely mapping reads). The total number of mappable reads for each agar plate culture control sample (hereafter referred as culture) ranged between 15.54 and 19.85 million reads. The number of detectable genes in culture samples ranged between 7763 and 7791, representing 93.93–94.23% of the total number of *Bd* genes. In the *A. zeteki* samples, between 23.66 and 48.18 million mappable reads were produced, of which 12.69–39.90% were identified as *Bd*. One of the four *H. lemur* samples produced <200,000 *Bd* reads (<1% of mappable reads); however, this frog carried by far the lowest *Bd* load (∼400 ZGE), indicating a lower limit to infection intensity for this LCM method. This sample was removed from the study, as it lacked sufficient coverage for subsequent analyses. The remaining three *H. lemur* samples had 44.77–59.28 million mappable reads, of which 6.70–8.15% were identified as *Bd*. All frog samples produced a similar number of detectable *Bd* genes compared to cultures (7234–7932, 87.60–95.94%), except sample HL3, in which we detected slightly fewer genes (6284, 76.00%). We observed no apparent correlation between host infection intensity and the percentage of *Bd* reads recovered in the remaining samples (Table 1). This is to be expected, as only regions of skin harboring clusters of sporangia were excised during LCM (see *Materials and Methods*).

Non-*Bd* reads from *A. zeteki* samples were mapped to a transcriptome previously characterized via standard Illumina RNAseq methods (Ellison *et al.* 2014). Of the 39,675 previously assembled transcripts, 54.25–77.52% were detected in this study. It should be noted that the previous assembly included sequence data from multiple tissues (whole skin, spleen, and small intestine), and so we would not expect to recover all genes in the current single tissue type (skin epidermis). In contrast, no transcriptome was available for *H. lemur*, therefore *de novo* assembly from our sequencing data were necessary. The *H. lemur* transcriptome consisted of 20,988 transcripts with blast hits (N50 = 797), of which 15,816 (75.36%) were functionally annotated. This is similar level of annotation to previous *de novo* assemblies of Neobatrachian transcriptomes (Ellison *et al.* 2015), albeit in an assembly with a somewhat lower N50 value. No uninfected host samples were available from this study; therefore, further simultaneous analyses of host responses (with *Bd* expression) were not possible, although we demonstrate this is feasible using our methodology.

### Bd gene expression

**Figure 1:**
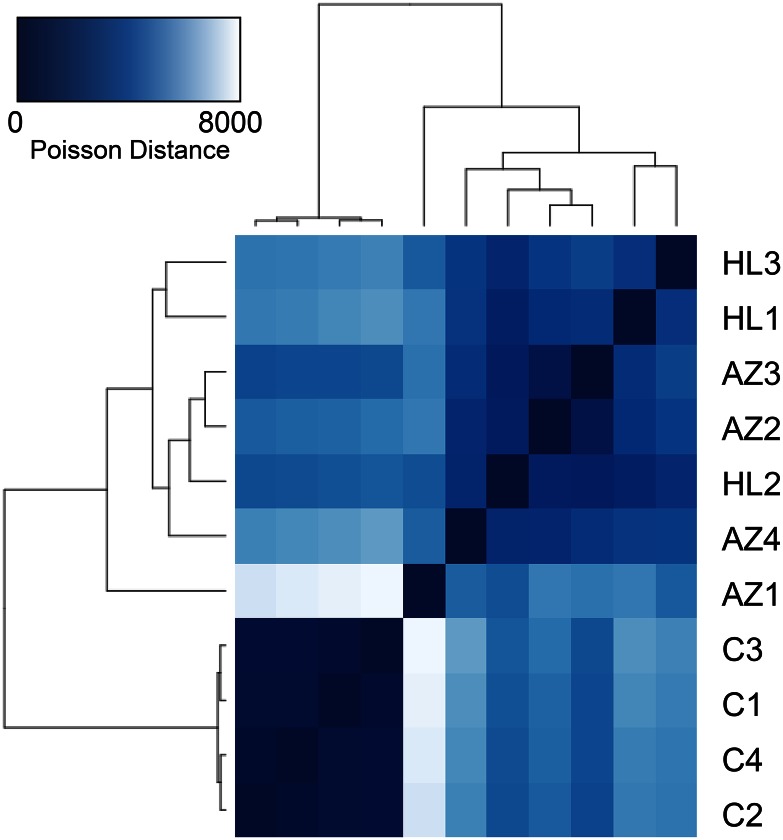
Heatmap of sample differences in gene expression. Based on hierarchical clustering of Poisson distances of *Bd* gene expression values. Color intensity indicates distance; darker blue equates to more similar samples. Sample letters indicate *Bd* treatment; *C* = agar culture plate, AZ = *A. zeteki*, and HL = *H. lemur*.

Hierarchical clustering of Euclidean distances between normalized *Bd* gene expression values revealed clear separation of *Bd* in culture and frog hosts (Figure 1). *Bd* culture samples were highly uniform in gene expression compared to frog samples. Moreover, at this broad level of expression profiles, *A. zeteki* and *H. lemur* derived *Bd* did not clearly segregate (Figure 1). Compared to cultured *Bd*, a total of 2190 and 2573 genes were significantly differentially expressed in *Bd* from *A. zeteki* and *H. lemur* hosts, respectively. Only 579 *Bd* genes were found to have significantly different expression levels between *A. zeteki* and *H. lemur* samples.

Significant enrichment of genes with increased expression in both host species compared to *Bd* cultures (*n* = 722) were assigned to GO terms related to proteolysis (*e.g.*, GO:0008236, “serine-type peptidase activity”), chitin binding (GO:0008061), and membrane transport activity (*e.g.*, GO:0003333, “amino acid transmembrane transporter activity”). *Bd* genes with lower expression in both host species compared to culture included GO terms related to protein kinases (*e.g.*, GO:0004674, “protein serine/threonine kinase activity”) and phosphorylation (*e.g.*, GO:0006468, “protein phosphorylation”). Full lists of significantly enriched GO terms are provided in Table 2.

**Table 2:** GO terms of genes found to be differentially expressed by *Bd* in culture and frog hosts

GO ID	GO Term	Category	FDR P-Value
Increased expression in both frog species			
GO:0008236	Serine-type peptidase activity	F	4.07E–05
GO:0017171	Serine hydrolase activity	F	4.07E–05
GO:0015171	Amino acid transmembrane transporter activity	F	2.89E–03
GO:0070011	Peptidase activity, acting on L-amino acid peptides	F	4.09E–03
GO:0008233	Peptidase activity	F	4.96E–03
GO:0008061	Chitin binding	F	5.36E–03
GO:0046943	Carboxylic acid transmembrane transporter activity	F	6.87E–03
GO:0005342	Organic acid transmembrane transporter activity	F	6.87E–03
GO:0008514	Organic anion transmembrane transporter activity	F	4.45E–02
GO:0004806	Triglyceride lipase activity	F	4.45E–02
GO:0003824	Catalytic activity	F	4.60E–02
GO:0006865	Amino acid transport	P	2.89E–03
GO:0003333	Amino acid transmembrane transport	P	2.89E–03
GO:0098656	Anion transmembrane transport	P	4.04E–03
GO:0006508	Proteolysis	P	4.09E–03
GO:0015849	Organic acid transport	P	3.17E–02
GO:0046942	Carboxylic acid transport	P	3.17E–02
GO:0055085	Transmembrane transport	P	3.90E–02
Decreased expression in both frog species			
GO:0004674	Protein serine/threonine kinase activity	F	1.89E–02
GO:0043167	Ion binding	F	1.89E–02
GO:0004672	Protein kinase activity	F	1.89E–02
GO:0003700	Sequence-specific DNA binding transcription factor activity	F	1.89E–02
GO:0001071	Nucleic acid binding transcription factor activity	F	1.89E–02
GO:0005488	Binding	F	3.26E–02
GO:0097159	Organic cyclic compound binding	F	4.57E–02
GO:1901363	Heterocyclic compound binding	F	4.57E–02
GO:0006468	Protein phosphorylation	P	1.89E–02
GO:0009069	Serine family amino acid metabolic process	P	3.86E–02

*F*, molecular function, *P*, biological process.

In comparisons of *Bd* gene expression in the two different hosts, 390 genes had higher expression levels in *A. zeteki*. These genes were enriched for terms related to cilia (*e.g.*, GO:0044782, cilium organization, GO:0060271, cilium morphogenesis). The 189 genes more expressed in *H. lemur Bd* samples were predominantly enriched for biosynthetic and amino acid metabolic processes (*e.g.*, GO:0044711, single-organism biosynthetic process, GO:0006573, valine metabolic process). Full lists of significantly enriched GO terms are provided in Table 3.

**Table 3:** GO terms of genes found to be differentially expressed by *Bd* in the two frog host species

GO ID	GO Term	Category	FDR P-Value
Increased expression in *Hylomantis lemur*			
GO:0003735	Structural constituent of ribosome	F	6.21E–07
GO:0005198	Structural molecule activity	F	1.41E–05
GO:0009058	Biosynthetic process	P	5.68E–09
GO:0044249	Cellular biosynthetic process	P	6.49E–09
GO:1901576	Organic substance biosynthetic process	P	6.85E–09
GO:0006412	Translation	P	4.23E–06
GO:0044237	Cellular metabolic process	P	5.31E–05
GO:0042254	Ribosome biogenesis	P	2.41E–04
GO:0071704	Organic substance metabolic process	P	2.74E–04
GO:0044238	Primary metabolic process	P	7.60E–04
GO:0022613	Ribonucleoprotein complex biogenesis	P	8.06E–04
GO:0044711	Single-organism biosynthetic process	P	1.14E–03
GO:0034645	Cellular macromolecule biosynthetic process	P	2.95E–03
GO:0009059	Macromolecule biosynthetic process	P	3.62E–03
GO:0009987	Cellular process	P	5.08E–03
GO:0044267	Cellular protein metabolic process	P	7.05E–03
GO:0006573	Valine metabolic process	P	1.52E–02
GO:0006551	Leucine metabolic process	P	1.52E–02
GO:0044085	Cellular component biogenesis	P	1.57E–02
GO:0008152	Metabolic process	P	2.48E–02
GO:0019538	Protein metabolic process	P	2.48E–02
GO:0006554	Lysine catabolic process	P	2.74E–02
GO:0009068	Aspartate family amino acid catabolic process	P	2.74E–02
GO:1901566	Organonitrogen compound biosynthetic process	P	2.75E–02
GO:0032787	Monocarboxylic acid metabolic process	P	2.75E–02
GO:0006414	Translational elongation	P	2.75E–02
GO:0018874	Benzoate metabolic process	P	3.03E–02
GO:0006549	Isoleucine metabolic process	P	3.36E–02
GO:0006574	Valine catabolic process	P	3.36E–02
GO:0006552	Leucine catabolic process	P	3.36E–02
GO:0006550	Isoleucine catabolic process	P	3.36E–02
GO:0044282	Small molecule catabolic process	P	3.86E–02
GO:0006082	Organic acid metabolic process	P	4.21E–02
GO:0009083	Branched-chain amino acid catabolic process	P	4.31E–02
GO:0009081	Branched-chain amino acid metabolic process	P	4.45E–02
GO:0044283	Small molecule biosynthetic process	P	4.87E–02
Increased expression in *Atelopus zeteki*			
GO:0005515	Protein binding	F	2.06E–03
GO:0044782	Cilium organization	P	4.21E–05
GO:0060271	Cilium morphogenesis	P	1.31E–04
GO:0042384	Cilium assembly	P	6.31E–04
GO:0010927	Cellular component assembly involved in morphogenesis	P	1.68E–03
GO:0030031	Cell projection assembly	P	1.68E–03
GO:0032990	Cell part morphogenesis	P	2.42E–03
GO:0048858	Cell projection morphogenesis	P	2.42E–03
GO:0030030	Cell projection organization	P	2.42E–03
GO:0048646	Anatomical structure formation involved in morphogenesis	P	8.69E–03
GO:0070925	Organelle assembly	P	8.69E–03
GO:0000902	Cell morphogenesis	P	2.16E–02
GO:0007224	Smoothened signaling pathway	P	2.17E–02
GO:0032989	Cellular component morphogenesis	P	2.73E–02

*F*, molecular function; *P*, biological process.

### Bd eSNV frequencies

**Figure 2:**
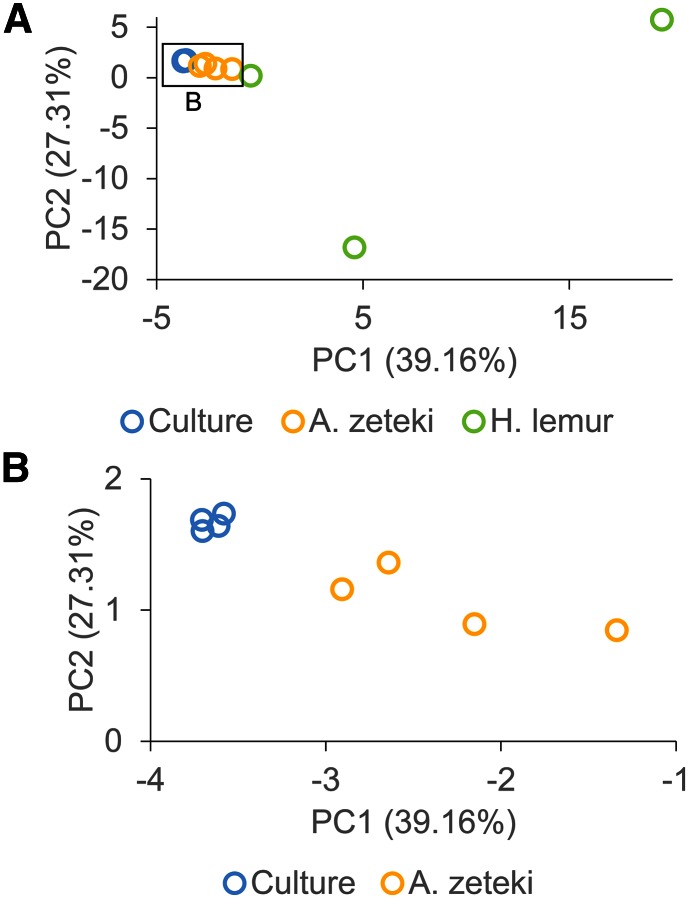
Separation of samples by eSNV frequencies. Principal component analysis of minor allele eSNV frequencies of *Bd*. Based on eSNVs with minor allele frequency (MAF) >0.1, minimum count = 6, minimum coverage = 20 (31,053 SNVs). (B) Depicts an enlarged area of (A).

Visualization of sample similarity based on all eSNV frequencies (31,053 sites) demonstrated clear separation of all three treatment groups (Figure 2). However, two of the three *H. lemur* samples showed a far greater degree of divergence than all other comparisons (Figure 2). A total of 25,575 biallelic SNVs passed our coverage, minor allele frequency (MAF) criteria, and frequency significance tests, resulting in 10,073 synonymous and 15,502 nonsynonymous changes. Of these, 12,262 (47.95%) had been previously identified as polymorphic sites during resequencing of the JEL423 genome (Farrer *et al.* 2013, File S3).

For the purpose of this study, we only considered differences in allele frequencies (*i.e.*, polymorphic sites present in all treatments but at different frequencies), and not new acquired SNVs (*de novo* mutations). We found 3863 eSNVs with significantly different allele frequencies between culture and *A. zeteki* hosts (synonymous; 1369, nonsynonymous; 2494), and, of these, 2570 (66.79%) had a higher MAF in *A. zeteki Bd* populations. We detected 20,276 significantly different expressed allele frequencies between culture and *H. lemur* groups (synonymous; 8591, nonsynonymous; 11,685); 17,516 (86.39%), of which had higher MAF in *H. lemur* infections. Comparing *A. zeteki* and *H. lemur* allele frequencies revealed 21,513 significant sites, with 17,619 of those with increased MAF in *H. lemur*. We found no significant enrichment of GO terms in genes carrying significantly different eSNV allele frequencies in any of the comparisons outlined above. Only a minor proportion (23.7%) of the genes harboring significantly different eSNV allele frequencies were found to be differentially expressed (Figure S2).

## Discussion

For the first time, we characterize the *in vivo* transcriptome of *Bd* within natural host tissues, providing exciting new theories on how changes in gene expression and infecting population genotypes can be key to the success of a generalist fungal pathogen. Gene expression of *Bd* differed substantially between standard laboratory culture conditions, and when infecting amphibian hosts. We also observed significant variation in *Bd* transcriptomes between two different amphibian host species. Finally, we found that *Bd* populations (measured as expressed genotype variant frequencies) were distinct between culture and amphibian skin, and between the two species of amphibian hosts.

Using laser-capture microdissection (LCM) of infected amphibian epidermal tissues, we captured the transcriptomes of both host and pathogen simultaneously via dual RNAseq. Though the majority of mappable reads were host-derived, this technique recovers sufficient *Bd* sequence data (ranging between 4 and 40% of mappable reads) to measure gene expression across the majority of the fungal transcriptome (>80% of genes). A previous study (Ellison *et al.* 2014) of highly infected skin samples only recovered 0.1–1% *Bd* reads using traditional RNAseq methods, therefore LCM offers a unique opportunity to comprehensively characterize *Bd* gene expression within hosts. Despite this improvement, our study also has some limitations. First, the lack of uninfected host samples precluded full simultaneous analysis of host responses alongside *Bd* gene expression. Second, as a developing methodology, our sample sizes were limited, and within-host variation of *Bd* via technical replicates controlling for host genotype were not characterized. Finally, to maximize opportunity to gather sufficient pathogen genetic material, we only sampled chytrid and host at a late stage of infection, thus missing early stages of the invasion dynamics. Nonetheless, our findings demonstrate the feasibility of studying interactions of *Bd* and host simultaneously at the cellular level, providing exciting new opportunities to understand this devastating amphibian pathogen. The next obvious application is to assess if this technique can capture earlier time points in the infection process to better understand how pathogen-host profiles are interlinked, and to characterize the *Bd*-amphibian “interactome.”

Over 2000 *Bd* genes were found to be significantly differentially expressed between culture and each host treatment. The 722 genes with higher expression in both host treatments compared to culture were enriched for functions related to proteolysis, particularly serine-type peptidases (Table 2). Moreover, a number of metallocarboxypeptidases were among the genes exhibiting the greatest increases in expression in both host treatments compared to culture (Table S1 and Table S2). Previous studies have shown expansion of these families of peptidases in the genome of *Bd* (Joneson *et al.* 2011; Rosenblum *et al.* 2013). In addition, *Bd* grown in broth enriched with pulverized frog skin exhibit increased gene expression of these classes (Rosenblum *et al.* 2012), and thus have been proposed as important for interactions with host tissues. However, while serine-type peptidase expression is thought to be predominantly invariant between *Bd* life stages, metallocarboxypeptidases are more highly expressed in sporangia than zoospores (Rosenblum *et al.* 2008). While our methods aimed to isolate sporangia in both culture and host samples, it is possible differences in number of zoospores per sporangia may contribute to our findings. Nonetheless, our results consolidate the hypotheses that serine-type and metallocarboxypeptidases are involved in amphibian host exploitation.

*Bd* transcripts with increased expression in amphibian hosts were also rich in genes related to membrane transport mechanisms (Table 2), with amino acid permeases being among those with greatest increases in expression (Table S1 and Table S2). In fungal plant pathogens, high expression of amino acid permeases is crucial for the acquisition of certain amino acids during the infection process (Solomon *et al.* 2003; Divon and Fluhr 2007). The overexpression of this class of transporters in *Bd* infecting amphibian hosts compared to culture could indicate important differences in nutrient availability, and/or higher requirement for exogenous amino acids, during invasion and proliferation within living host tissues. Currently it is unknown the action and specificity of these amino acid transporters in *Bd*, and thus functional characterization is required to test this hypothesis. Another group of transporters with large increases in expression in *in vivo Bd* transcriptomes were annotated as putative major facilitator superfamily (MFS) members (Table S1 and Table S2). In other pathogenic fungi MFS transporters are important in the active secretion of toxic compounds (Del Sorbo *et al.* 2000). This, and the recent discovery that *Bd* secretes a lymphocyte inhibitory factor (Fites *et al.* 2013), suggests these MFS transporters highly expressed during host infection are likely candidates for *Bd* virulence. Alternatively, they could contribute to *Bd* defense mechanisms as pumps of toxins produced by either host or its microbiome, similar to drug efflux pumps in other animal fungal pathogens (Morschhäuser 2010). In either case, these MFS member warrant further characterization as likely key to *Bd* survival within host.

*Bd* genes sharing increased expression in both host species compared to culture were also enriched for the GO term “chitin binding” (Table 2). Chitin deacetylases were among those transcripts with the greatest increases of expression in both frog treatments (Table S1 and Table S2). Chitin-binding proteins and chitin deacetylation provide fungal pathogens with protection against host chitinase defense responses (El Gueddari *et al.* 2002; van den Burg *et al.* 2006). Intriguingly, the *Bd* genome appears to have undergone an expansion of genes encoding chitin-binding proteins (Abramyan and Stajich 2012), and *Bd* recombinant chitin-binding proteins confer the fungus, *Trichoderma reeseii*, *in vitro* protection against chitinase activity (Liu and Stajich 2015). On the host side, chitinase production appears to be an important *Bd* defense mechanism in amphibians; individuals exhibiting greater expression levels are more likely to clear *Bd* infections (Ellison *et al.* 2014, 2015). The overexpression of *Bd* chitin deacetylases and other chitin-binding proteins in live host infections compared to culture indicates they are likely a critical protective mechanism for *Bd*, thus further work to confirm the causal link of their expression and function against host chitinase will be a productive direction for future research.

The broad-scale gene expression profiles of *Bd* in our two focal host species were very similar (Figure 1); nonetheless, we found 579 *Bd* transcripts differentially expressed between *A. zeteki* and *H. lemur* hosts. Genes more highly expressed in *A. zeteki* hosts were enriched for many GO terms related to flagella (Table 3), suggesting a greater investment in motile zoospore production. This finding is consistent with the fact that *A. zeteki* is an acute “supershedder” of *Bd* zoospores (DiRenzo *et al.* 2014). In contrast, genes more highly expressed in *H. lemur* infections were enriched for a number of biosynthetic and catabolic processes (Table 3). Earlier studies in culture show that abiotic conditions can significantly alter life history trade-offs in *Bd* (Woodhams *et al.* 2008). Our results indicate that host species also influences the relative investment of *Bd* in growth and reproduction, likely as a response to the host’s defensive capabilities (Ellison *et al.* 2015). However, though our methods strive to sample equivalent life stages in all samples, we cannot rule out the possibility microdissection of infected tissues may lead to stochastic differences in *Bd* cell types captured. Nonetheless, for a generalist such as *Bd*, the selective environment of different host species is expected to have a strong effect, and our data indicates that the pathogen may respond by shifts in resource allocation rather than evolutionary changes. This level of flexibility is most likely part of the strategy ultimately underlying the success of *Bd* as a generalist pathogen.

Across the *Bd* transcriptome, we found a substantial number of single-nucleotide variants with significantly different allele frequencies among culture and the two host species. Our RNAseq data do not allow us to discriminate whether these allele frequency differences are due to allele-specific expression, or underlying genotype frequency differences in the *Bd* populations (Konczal *et al.* 2014); therefore, we refer only to “expressed genotypes.” Our data show a clear shift in expressed genotypes from culture to amphibian host (Figure 2). This was particularly striking in *H. lemur* hosts, where the majority of *Bd* eSNVs, rare in culture, are more prevalent in amphibian skin. *Bd* typically attenuates in the relaxed or altered selective environment of artificial culture media (Berger *et al.* 2005; Retallick and Miera 2007; Langhammer *et al.* 2013). However, *Bd* can also regain virulence after only a single passage through an amphibian host (Brem *et al.* 2013). Our data show that these rapid changes in pathogenicity may be mediated by equally rapid changes in population expressed genotype frequencies, either via the relative success and survival of particular sequence variants in living hosts or via allele-specific expression. Yet we did not find any consistent pattern of differential expression within genes harboring significant eSNV frequencies. For example, only a small proportion of genes with higher minor allele frequencies in *H. lemur* also exhibited higher expression, or vice versa (Figure S2). We also found no functional enrichment of genes harboring significantly different eSNVs, perhaps unsurprising as this is only a single host passage to select upon genetic variation within the *Bd* culture stock used for these experiments. We cannot determine from our data if recombination and/or sexual reproduction has occurred *in vivo*, causing increases in some allele frequencies as a result of hitch-hiking with selected alleles, which would confound functional enrichment analyses. However, as a single strain was used for this study, and no sexual recombination has been directly observed for any population of *Bd*, this seems unlikely.

Anueploidy is a significant feature of chytrid genomes (Farrer *et al.* 2013; Rosenblum *et al.* 2013), and rapid changes in *Bd* virulence during serial passages through culture has been linked to shifts in chromosome copies (Refsnider *et al.* 2015). This has the potential to influence both gene expression (Galitski *et al.* 1999) and genotype frequency data (Rosenblum *et al.* 2013), and may in part explain differences observed here. Thus, future studies should incorporate our methods with DNA data (to disentangle sequence variation, allele-specific expression, recombination, and aneuploidy), and multiple passages through living hosts. This will undoubtedly uncover the relative contributions of gene expression regulation and ploidy levels, and ultimately provide insights on whether these changes underlie the observed rapid shifts in *Bd* virulence and their mechanisms.

Our characterization of the first *in vivo* transcriptomes of *Bd* from natural host skin provides new insights into host-pathogen interactions and mechanisms underlying rapid changes in virulence and resistance to host defenses. Our findings support earlier hypotheses that secreted proteases are vital for mechanisms of host tissue exploitation (Joneson *et al.* 2011; Rosenblum *et al.* 2012, 2013). Our data imply expression of particular classes of transmembrane transporters as potentially important for acquisition of host-derived nutrients, and others likely involved in secretion of virulence factors. We provide the first *in vivo* association of chitin deacetylase production with *Bd* within-host defense mechanisms. *Bd* gene expression is highly host specific, resulting in host-dependent differences in fungal allocation to growth and reproduction. Finally, we identified significant shifts in *Bd* expressed genotypes between culture and host, and between host species. Our findings have broader implications for chytrid transmission in wild amphibian populations. If passage through a particular host species results in substantial shifts in *Bd* expressed genotypes and life-history trait investment, the composition of any amphibian community may result in important changes in the infection dynamics of *Bd*. This provides a compelling explanation for observed changes in *Bd* virulence during its spread southwards through Central America (Phillips and Puschendorf 2013).

Our results highlight the urgent need for in-depth functional studies of *Bd* and other emerging pathogens within natural hosts. We show that changes of the *Bd* gene expression repertoire, influenced by host species, have potential to dictate amphibian–chytrid interactions from the cellular level of infection and exploitation, to transmission dynamics via changes in resource allocation, and through the community level, as host species composition shapes *Bd* populations. Only by elucidating this intricate interplay of host and pathogen on this level will we fully understand how this emergent pathogen persists and thrives in amphibian communities worldwide.

## Supplementary Material

Supplemental material is available online at www.g3journal.org/lookup/suppl/doi:10.1534/g3.116.035873/-/DC1.
