# Supplementary material for: First in Vivo Batrachochytrium dendrobatidis Transcriptomes Reveal Mechanisms of Host Exploitation, Host-Specific Gene Expression, and Expressed Genotype Shifts
Source: G3 (Bethesda). 2016 Nov 16;7(1):269–78. doi: 10.1534/g3.116.035873 (PMC5217115; doi:10.1534/g3.116.035873)
Supplement: Supplementary file 1 [file 269FigureS1.pdf]

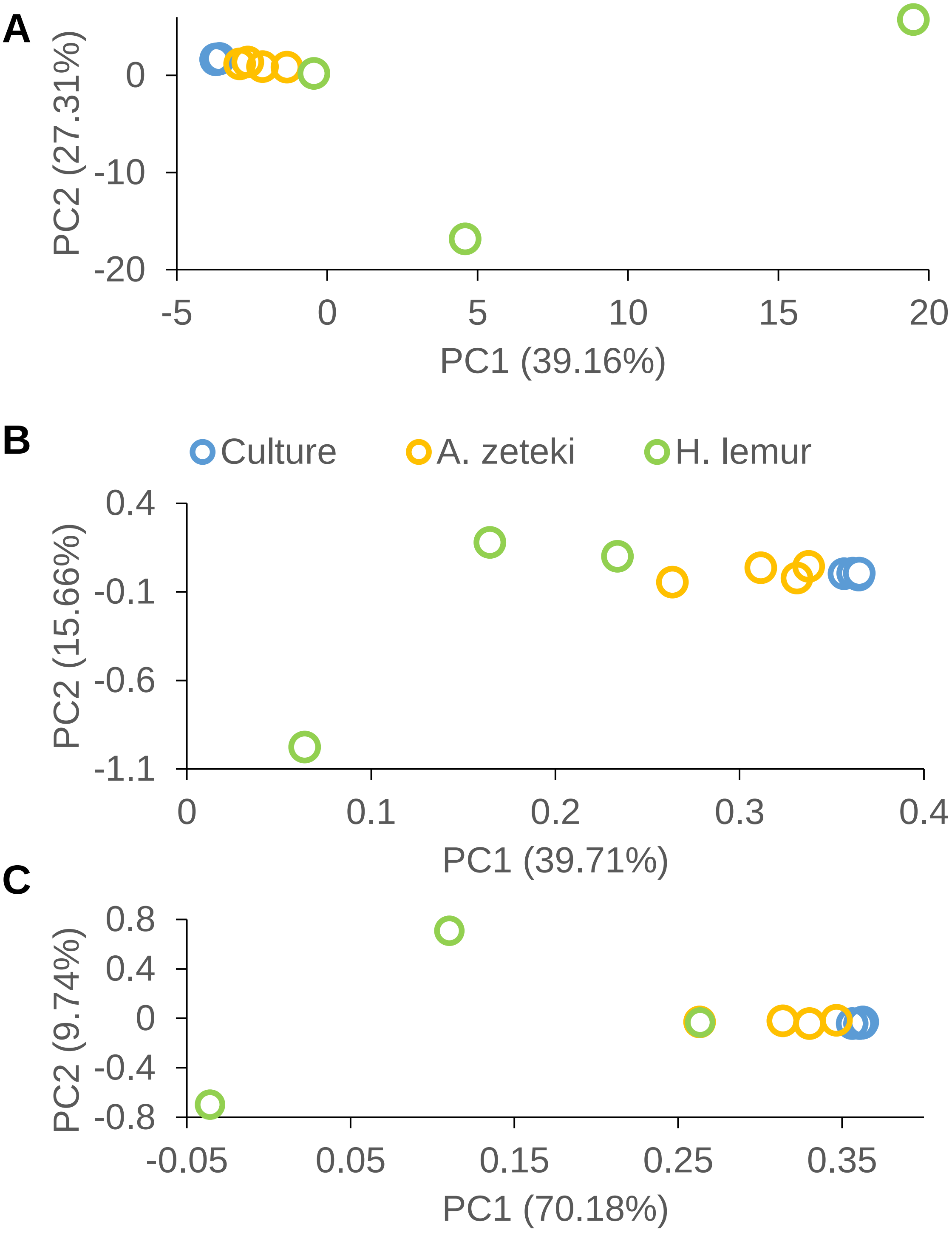

Figure S1. Separation of samples by eSNV frequencies. Principal component analysis of minor allele SNP frequencies. Based on SNPs with minor allele frequency (MAF) >0.1, minimum coverage = 20, and A) minimum count = 6 (31,054 SNVs), B) minimum count = 10 (12,271 SNVs), or C) only SNPs previously found by genome resequencing (Farrer et al. 2013) with minimum count = 6 (12,262 SNVs).
