# Supplementary material for: First in Vivo Batrachochytrium dendrobatidis Transcriptomes Reveal Mechanisms of Host Exploitation, Host-Specific Gene Expression, and Expressed Genotype Shifts
Source: G3 (Bethesda). 2016 Nov 16;7(1):269–78. doi: 10.1534/g3.116.035873 (PMC5217115; doi:10.1534/g3.116.035873)
Supplement: Supplementary file 3 [file 269FileS1.docx]

File S1: Summary of all differential gene expression tests. (.xls, 3.68 MB)

Available for download as an .xls file at

<http://www.g3journal.org/lookup/suppl/doi:10.1534/g3.116.035873/-/DC1/FileS1.xls>
