# Supplementary material for: First in Vivo Batrachochytrium dendrobatidis Transcriptomes Reveal Mechanisms of Host Exploitation, Host-Specific Gene Expression, and Expressed Genotype Shifts
Source: G3 (Bethesda). 2016 Nov 16;7(1):269–78. doi: 10.1534/g3.116.035873 (PMC5217115; doi:10.1534/g3.116.035873)
Supplement: Supplementary file 5 [file 269FileS2.docx]

File S2. All eSNVs with significant differences in frequencies and their transcript locations. (.csv, 2.56 MB)

Available for download as .csv at <http://www.g3journal.org/lookup/suppl/doi:10.1534/g3.116.035873/-/DC1/FileS2.csv>
