# Supplementary material for: First in Vivo Batrachochytrium dendrobatidis Transcriptomes Reveal Mechanisms of Host Exploitation, Host-Specific Gene Expression, and Expressed Genotype Shifts
Source: G3 (Bethesda). 2016 Nov 16;7(1):269–78. doi: 10.1534/g3.116.035873 (PMC5217115; doi:10.1534/g3.116.035873)
Supplement: Supplementary file 7 [file 269FileS3.docx]

File S3. Details of the eSNVs matched to previous resequencing studies and their genomic locations. (.xls, 3.43 MB)

Available for download as .xls at http://www.g3journal.org/lookup/suppl/doi:10.1534/g3.116.035873/-/DC1/FileS3.xls
