# Supplementary material for: First in Vivo Batrachochytrium dendrobatidis Transcriptomes Reveal Mechanisms of Host Exploitation, Host-Specific Gene Expression, and Expressed Genotype Shifts
Source: G3 (Bethesda). 2016 Nov 16;7(1):269–78. doi: 10.1534/g3.116.035873 (PMC5217115; doi:10.1534/g3.116.035873)
Supplement: Supplementary file 9 [file 269FileS4.docx]

File S4. GO annotation of the JEL423 reference transcriptome generated by Blast2Go. (.zip, 119 KB)

Available for download as a .zip file at

File S4: http://www.g3journal.org/lookup/suppl/doi:10.1534/g3.116.035873/-/DC1/FileS4.zip
