# Supplementary material for: First in Vivo Batrachochytrium dendrobatidis Transcriptomes Reveal Mechanisms of Host Exploitation, Host-Specific Gene Expression, and Expressed Genotype Shifts
Source: G3 (Bethesda). 2016 Nov 16;7(1):269–78. doi: 10.1534/g3.116.035873 (PMC5217115; doi:10.1534/g3.116.035873)
Supplement: Supplementary file 11 [file 269FileS5.docx]

File S5. Custom perl script used to determine synonymous and non-synonymous eSNVs. (.zip, 2.60KB)

Available for download as a .zip file at

File S4: http://www.g3journal.org/lookup/suppl/doi:10.1534/g3.116.035873/-/DC1/FileS5.zip
