# Supplementary material for: First in Vivo Batrachochytrium dendrobatidis Transcriptomes Reveal Mechanisms of Host Exploitation, Host-Specific Gene Expression, and Expressed Genotype Shifts
Source: G3 (Bethesda). 2016 Nov 16;7(1):269–78. doi: 10.1534/g3.116.035873 (PMC5217115; doi:10.1534/g3.116.035873)
Supplement: Supplementary file 13 [file 269TableS1.docx]

Table S1. Top 20 differentially expressed *Bd* genes comparing culture and *Atelopus zeteki*. (.pdf, 12.5 KB)

Available for download as a .pdf file at <http://www.g3journal.org/lookup/suppl/doi:10.1534/g3.116.035873/-/DC1/TableS1.pdf>
