# Supplementary material for: First in Vivo Batrachochytrium dendrobatidis Transcriptomes Reveal Mechanisms of Host Exploitation, Host-Specific Gene Expression, and Expressed Genotype Shifts
Source: G3 (Bethesda). 2016 Nov 16;7(1):269–78. doi: 10.1534/g3.116.035873 (PMC5217115; doi:10.1534/g3.116.035873)
Supplement: Supplementary file 2 [file 269FigureS2.pdf]

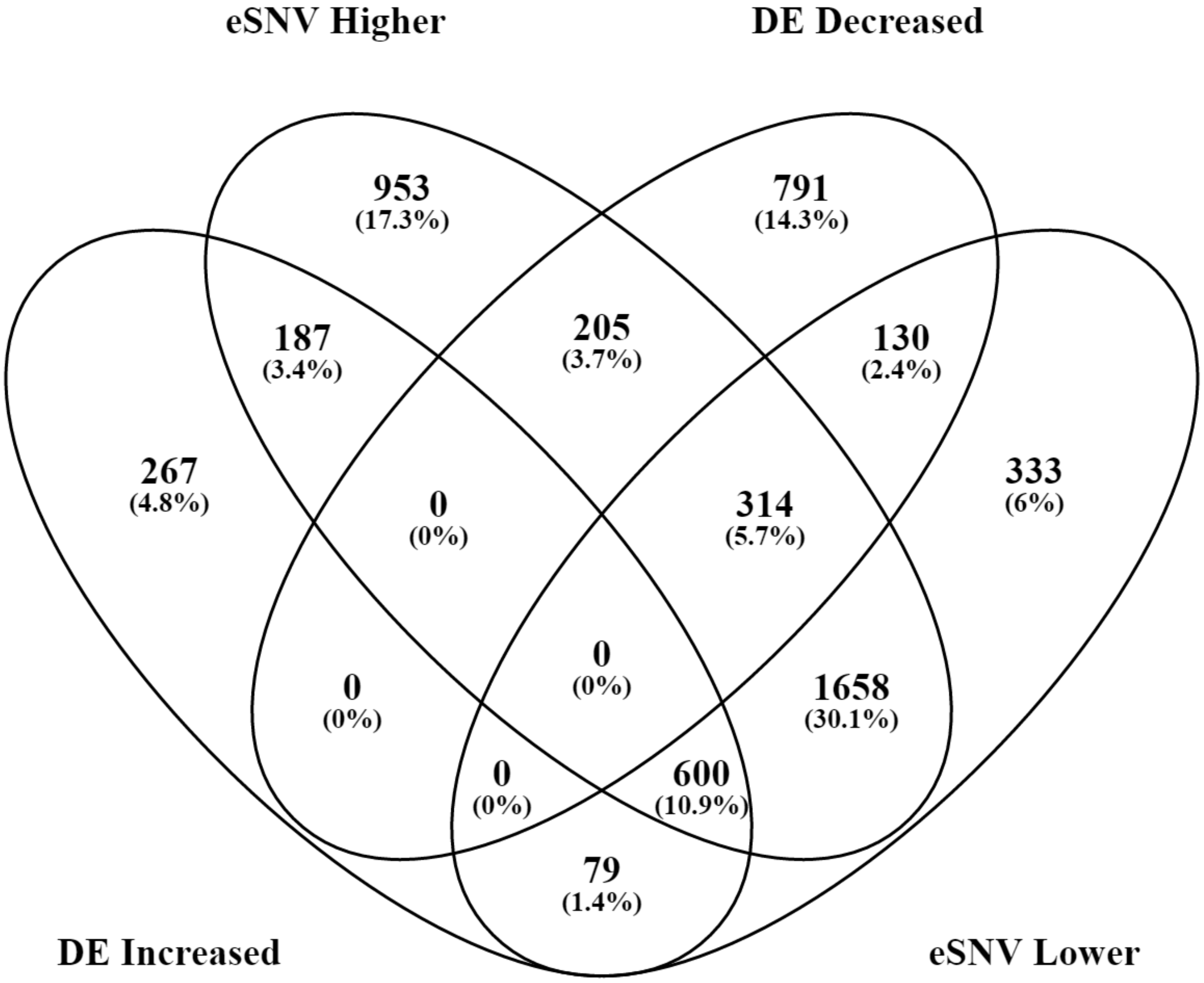

Figure S2. Overlap of differentially expressed genes and eSNVs. Venn diagram showing overlap of differentially expressed genes and eSNV frequencies of JEL423 in *Hylomantis lemur* compared to culture.
